# Optically-Directed Bubble Printing of MXenes on Flexible Substrates toward MXene-Enabled Wearable Electronics and Strain Sensors

**DOI:** 10.1021/acs.nanolett.4c06355

**Published:** 2025-04-16

**Authors:** Marcel Herber, Eric H. Hill

**Affiliations:** † Institute of Physical Chemistry, University of Hamburg, Grindelallee 117, 20146 Hamburg, Germany; ‡ The Hamburg Center for Ultrafast Imaging (CUI), Luruper Chaussee 149, 22761 Hamburg, Germany

**Keywords:** directed assembly, nanoparticle assembly, laser printing, MXene patterning, strain sensing, flexible electronics

## Abstract

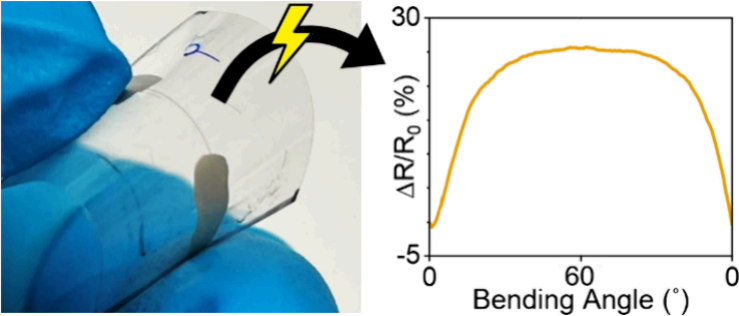

This study presents the use of laser-driven microbubbles for micropatterning Ti_3_C_2_T_X_ MXenes on flexible polyethylene terephthalate films, yielding conductive micropatterns without the need for pre- or postprocessing. Characterization of the electrical properties under varying strain conditions revealed distinct responses; resistance decreased under compressive strain and increased under tensile strain, demonstrating their potential as strain sensors. The patterns maintained functional integrity over 1000 cycles of bending, with a significant increase in resistance observed under tensile strain (61.6%) compared to compressive strain (11.3%). In addition, narrower MXene lines exhibited greater strain sensitivity, while broader lines were more robust. This work underscores the potential of bubble printing as an effective approach for printing conductive micropatterns and emphasizes its potential for substantial advances in wearable technology, flexible electronics, and strain sensing technologies.

MXenes are a relatively new class of two-dimensionally materials, discovered in 2011 by Gogotsi and co-workers.^1^ They are described by the general formula M_*n*+1_X_*n*_T_X_, where M represents a transition metal (e.g., Ti, Nb, V), X is C and/or N and T stands for the surface termination (typically O, OH, F). MXenes have generated great interest in recent years due to their unique properties. MXenes show high mechanical strength,^2,3^ tailorable optical properties,^4,5^ high electrical conductivity,^6−8^ biocompatibility,^9,10^ ionic conductivity and storage capacity,^11,12^ and blocking of electromagnetic fields in the microwave regime.^13,14^ Thus, MXenes have been used for applications in many different fields, including optoelectronics,^15^ energy storage,^11,12,16^ medicine,^9,10,17^ shielding against electromagnetic interference,^13,14^ and sensing.^18−20^ Taking advantage of the high mechanical strength and electric conductivity of MXenes, strain sensing using MXene-based composites has been reported,^19−22^ however so far these have been mostly in the form of films or bulk composites.

The freeform printing of MXenes into micrometric patterns can provide flexibility to approach different designs and integrated devices. There are several existing approaches for micropatterning, such as inkjet printing,^15,23^ screen printing,^24,25^ or direct ink writing.^26,27^ However, these techniques have a number of limitations, such as lower resolution (around 50 μm averaged across), lack of broader material compatibility, low throughput, and the need for pre/post-processing. Recently, the use of laser-induced microbubbles to attract and deposit matter on solid surfaces has been shown to be a simple and effective approach for patterning materials on solid surfaces.^28−31^ Henceforth referred to as “Bubble printing”, much of the early work on this method utilized plasmonic films for the photon-phonon conversion of light to heat in order to control bubble formation.^32−35^ However, this intrinsically limits applications where the printing of conductive materials is required, as electrically conductive plasmonic metals such as gold may short the circuit. In this direction, the bubble printing of silver nanoparticles and conductive polymers directly onto glass substrates has been achieved,^36,37^ however both of these require postprocessing in order to achieve good conductivity. Recently, our group has overcome the need for a plasmonic film on the substrate by directly heating colloids such as gold nanoparticles and MXenes, allowing their printing on arbitrary substrates.^38,39^ However, applications such as wearable electronics and strain sensors would require printing on common flexible substrates, which has not yet been achieved.

In this work, we report the bubble printing of Ti_3_C_2_T_X_ MXene on flexible films of the common plastic polyethylene terephthalate (PET), providing conductive micropatterns without any pre- or postprocessing steps. The electronic properties of the printed micropatterns were characterized, and the influence of bending on their electrical conductivity was measured, providing an understanding of their electrical response under different degrees of tensile and compressive strain as well as their longevity over repeated bending cycles. The printed MXene lines maintained their functional integrity even during deformation, with a minimal change in resistance observed during compressive strain. Interestingly, tensile strain led to a marked change in the resistance of the printed micropatterns, revealing their promise as micropatterned strain sensors. Thus, the bubble printed conductive MXene micropatterns are suitable for applications in both strain sensing and flexible electronics. Moreover, the small size and facile preparation of such patterns is ideal for integration into compact electronic devices and applications. Overall, the successful adaptation of bubble printing of MXenes onto flexible substrates paves the way for their use in wearable tech, electronic sensing, and lab-on-chip devices.

As described in detail in the methods section (see Supporting Information), Ti_3_C_2_T_X_ MXene was synthesized by etching away the Al from Ti_3_AlC_2_ MAX phase via the in situ generation of hydrofluoric acid in a solution of lithium fluoride and hydrochloric acid.^40^ The synthesized Ti_3_C_2_T_X_ MXene exhibits characteristic plasmon band at 741 nm, and electron microscopy reveals a typical flake-like morphology (Figure 1a). Bubble printing was carried out by focusing a laser onto the interface between a PET substrate and the colloidal dispersion of the MXene with an inverted optical microscope (Figure 1b).

**Figure 1:**
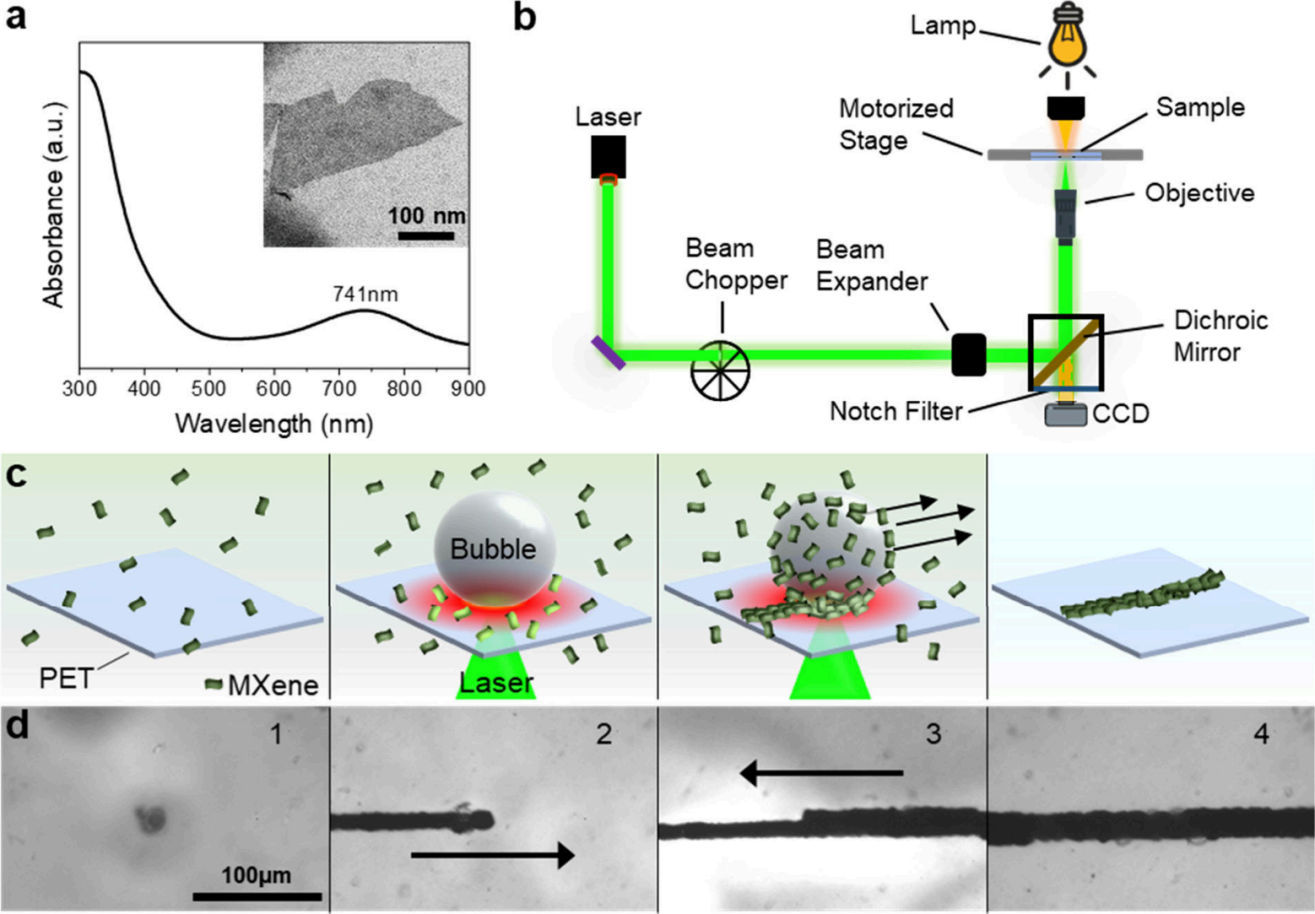
(a) UV–vis spectrum of synthesized Ti_3_C_2_T_X_ MXene, with a TEM image of a MXene flake inset; (b) Scheme of the optical setup used for bubble printing; (c) Scheme of the process of bubble printing; (d) Microscope images of bubble printing of a MXene line: (1) laser on, (2) movement to the right, (3) movement to the left, (4) final printed pattern. A typical example corresponding to (d) is shown in Video S1.

The process of bubble printing of MXene-based patterns is shown in Figure 1c, and is described as follows: (1) MXene dispersion is pipetted on the PET film. (2) The laser is focused on the substrate–liquid interface, and the heating induced by absorption of light by the MXene nanosheets heats the solvent to form a microbubble at the substrate/liquid interface. The surface tension gradient caused by the formation of the bubble induces Marangoni convection, causing colloidal particles to be drawn toward the microbubble. (3) After encountering the bubble interface, the MXene particles become immobilized on the substrate at the triple contact line, due to a combination of forces, especially capillary and van der Waals (VdW) interactions.^32^ The movement of the microscope stage then provides the capability to print arbitrary patterns of assembled MXene particles. The adhesion of the MXene patterns onto the PET substrate is realized without the need for functionalization of the glass substrate, which may be due to melting and cooling of the PET where MXene is deposited. (4) Bubble printed MXene patterns on the PET substrate are ready for use after being rinsed with water and dried under compressed air.

A typical printed MXene line with a width of 21 μm was fabricated by printing two lines by moving the stage in a linear path in opposite directions with a slight overlap between the two (Figure 1d). Scanning electron microscopy (SEM) revealed a largely smooth surface with many random holes with sizes ranging from hundreds to tens of nanometers. Such holes are likely formed by vapor escaping from the inside of the patterns during printing. While such holes have been previously observed, the increased resolution of these images compared to previous findings reveal “wall” and “bridge” type structures that have been previously observed in freeze-dried MXenes (Figure 2a, bottom panel).^41^ These observations suggest both densely packed stacked assemblies and porous structures due to the percolation of vapor from the assembly during printing, which can lead to alignment of the resulting porous channels along the curvature of the pattern.^42^ Moreover, SEM revealed that after 1000 cycles of bending to 60.3° under tensile strain the printed patterns showed visible signs of damage (Figure 2b). Certain regions of the printed lines showed visible cracks (Figure 2b, middle panel), and some segments appeared to have flaked off (Figure 2b, bottom panel), however, no complete breakage was observed. Cross-sectional SEMs provided significant insight into the adhesion of the bubble-printed MXene patterns and the underlying PET substrate (Figure S3). The cross-sectional view of MXene layers revealed the layered arrangement achieved through the printing process (Figure S3a). Interestingly, holes in the PET substrate beneath the printed MXene was observed, likely resulting from localized melting during MXene deposition (Figure S3b). A close-up view of the interface between the MXene and PET layers shows a clear transition between the MXene flakes at the surface, the PET substrate at the base, and an intermediate layer suggestive of melted PET (Figure S3c). This indicates that the robust adhesion of the MXene patterns is driven in part by heat-induced bonding.

**Figure 2:**
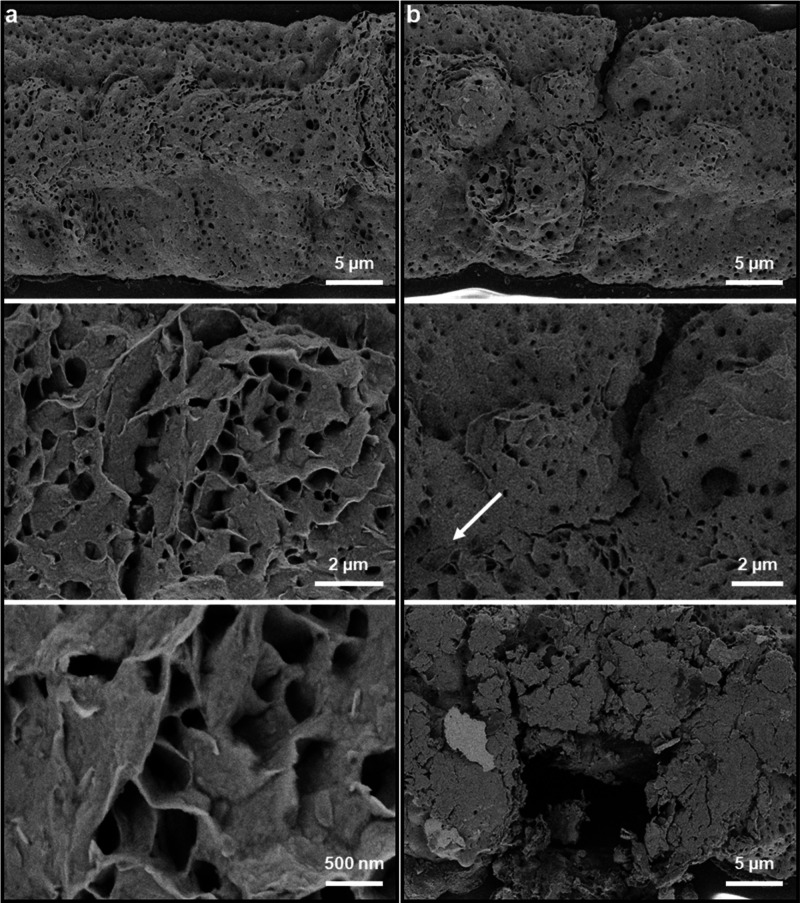
Scanning electron micrographs of bubble printed MXene patterns (a) without applied strain and (b) after 1000 applied to cycles of bending to 60.3° under tensile strain, showing microcracks and flaking damage. The white arrow in (b) indicates the extent of a microcrack. Additional images are given in Figure S2.

Lines of ∼7 mm (the diameter of the imaging spacer used) were printed, and then contacted by applying silver paste to the ends of the lines (Figure 3a). A custom-built bending setup was used to control the bending direction and angle of the MXene lines printed on PET (Figure 3b). Bending was applied in two different directions: toward the surface that the line was printed on (compression) and in the direction away from the printed side (decompression). This differentiates between the type of strain that is applied to the sample. Under compression the sample undergoes compressive strain, which is defined by the measure of how much a material shortens or deforms when subjected to compressive forces. Under decompression, the sample undergoes tensile strain, which is how much a material elongates or deforms when subjected to tensile forces.^43^

**Figure 3:**
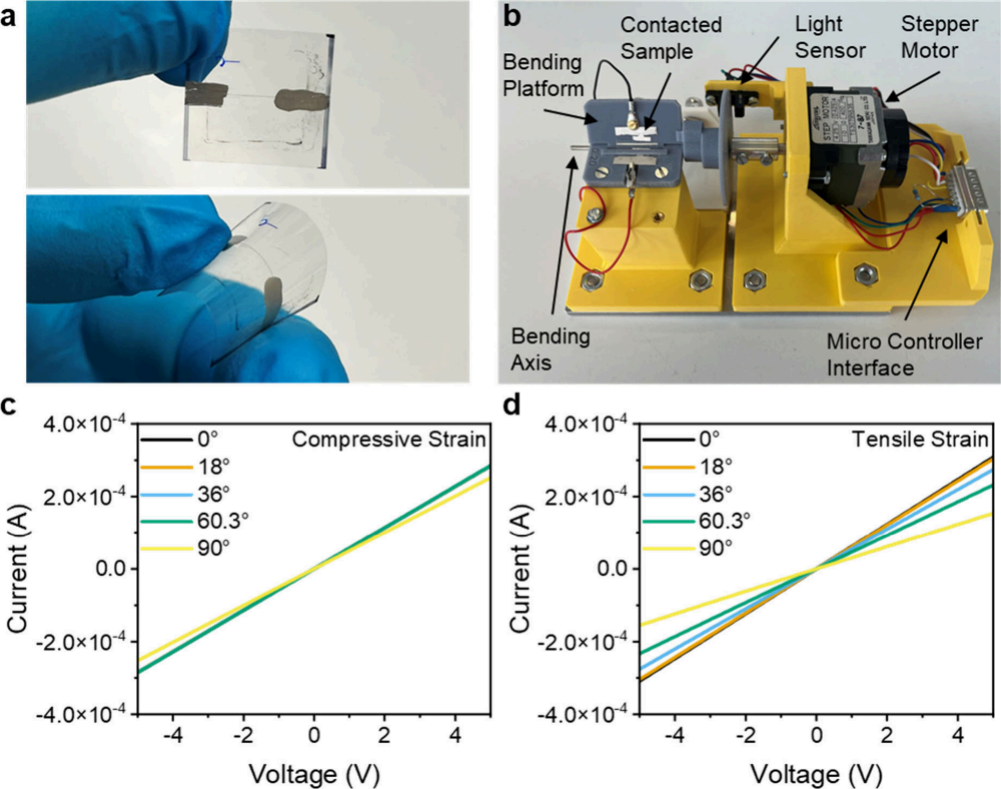
(a) Photograph of the bubble printed MXene pattern on PET film unbent (top) and bent (bottom); (b) Photograph of the custom-built bending setup; IV curves of MXene patterns at different bending angles for applied (c) compressive and (d) tensile strain. A zoom of (c) can be found in Figure S4.

The current–voltage (*IV*) curves of the bubble printed MXene patterns were measured at different bending angles in both directions in order to compare how compressive and tensile strain influence the electronic properties. The *IV*-characteristics demonstrated linear behavior in the range of −5 to 5 V, showing ohmic conductivity, with a resistance of 6.9 kΩ for unbent samples. There were marked differences in the current–voltage curves under compressive and tensile strain. Under compressive strain there is almost no change in resistance up to a bending angle of 60.3°, with a mean resistance of 17.6 kΩ, where increasing bending to 90° increases the resistance to 19.8 kΩ (Figure 3c). In contrast, when tensile strain is applied (Figure 3d), there is an increase in resistance for bending from 0°, with 16.2 kΩ, to 90°, with 32.4 kΩ. This marked difference between the current–voltage characteristics indicates that the bubble printed MXene patterns are much more sensitive to tensile than to compressive strain, where the observed change in resistance is attributed to physical deformation or microstructural changes (i.e., microcrack formation)^44^ resulting in decreased effective contact between the MXene particles that limits the electron flow (Figure 2b). These results are in line with typical responses from strain sensors, where under compressive strain the resistance decreases and under tensile strain the resistance increases.^45−47^

The stability of the conductive MXene micropatterns during repeated bending was tested by measuring the change in resistance of the printed line over 1000 cycles of repeated bending in both directions (Figure 4a,b). Under bending to 60.3° in the compressive direction, there was an average increase in resistance of 11.3% across all samples, where the maximum increase in resistance for a single sample was 25.3% (Figure 4c). However, the average increase in resistance over 1000 cycles of tensile bending was 61.6%, which shows that the samples subjected to compressive strain have a higher overall stability, with a 5.5 times lower change in resistance (11.3%). Thus, bubble printed MXene patterns exhibited greater longevity when subjected to compressive strain rather than tensile strain.

**Figure 4:**
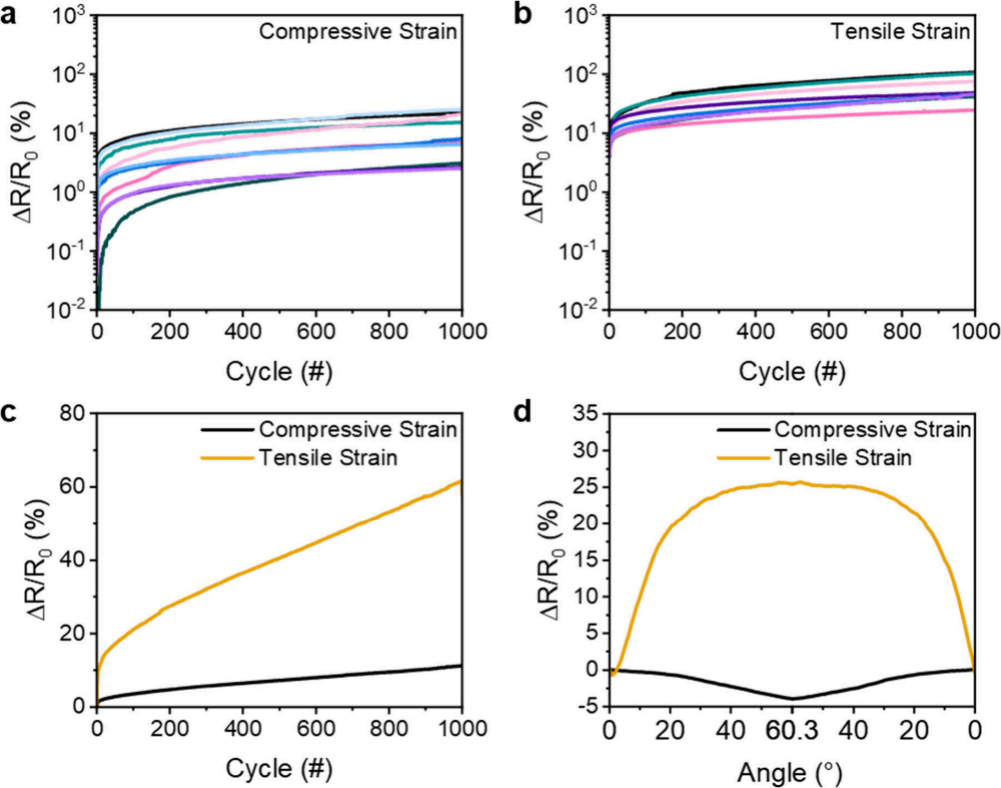
Cycle stability of bubble printed MXene lines under repeated cycles of bending from 0° to 60.3° to apply (a) compressive and (b) tensile strain. (c) Average change in resistance for samples shown in (a) and (b) over 1000 cycles in both bending directions. (d) Average change in resistance within a single cycle of bending to 60.3° after 999 cycles.

Moreover, the change in electrical resistance of the bubble printed MXene patterns under compressive and tensile strain after 999 cycles revealed distinct responses. As shown in Figure 4d, the sensor exhibited an average decrease in resistance of −4.0% with 60.3° of bending under compressive strain. This observation is typical of conductive films, and generally indicates that the distance between conductive particles decreases during compression, leading to an increase in electrical conductivity. In contrast, when subjected to tensile strain, the printed MXene lines demonstrated an averaged increase in resistance of 25.7%. This increase in resistance can be explained by a larger distance between MXene particles as tension is applied, and is a typical response profile for resistive strain sensors.^45−47^

In addition to the different overall change in resistance upon compressive and tensile strain, the change of resistance with increasing bending angle is also different depending on whether compressive or tensile strain is applied (Figure 4d). Under compressive strain, the resistance decreases in a mostly linear fashion from 0 to 60.3°. However, in the case of tensile strain, the resistance increases linearly by ∼17.1% from a bending angle of 0° to ∼16°, where the resistance increases only an additional 8.4% between ∼16° and 60.3°. This suggests that strain sensors fabricated by this approach would be most sensitive in the range between 0° and ∼16° bending, where the responsivity at bending angles between 16° and 60° is 5.5 times lower. The strong difference in the resistive behavior between compressive and tensile strain highlights the inherent anisotropic electronic behavior of the bubble printed MXene lines, since they are only deposited on one side of the PET film, and supports their potential for applications in both flexible electronics and strain sensors.

There was a marked change in the response within the first bending cycle compared to after 999 cycles (Figure S5b). The first bending led to an average change in resistance of – 1.7% with 60.3° of bending under compressive strain, 2.4 times lower responsivity than after 999 cycles of bending (−4.0%). Bending in the tensile direction led to an average increase in resistance of 15.0%, 1.7 times lower than after 999 cycles of bending (25.7%). Furthermore, under tensile strain there was relatively high hysteresis within the first bending cycle, with a change in resistance of 7% from start to end of the measurement, whereas the resistance within the 1000th cycle of bending showed low hysteresis (<0.4%).

While the MXene lines discussed above have a width of 21 μm, the influence of the line width on electrical properties under compressive strain was studied by comparing with lines with a width of 40 μm. With 40 μm lines, a change of 1.5% in resistance after 1000 cycles of compressive strain was measured (Figure S6a). This value for the 40 μm lines is ∼ 7.5 times lower than for lines with a width of 21 μm (11.3%). Furthermore, the 40 μm lines show a change of resistance within a single bending cycle of – 0.8% under compressive strain, which is around 5 times lower than the single cycle change in resistance for 21 μm lines (−4.0%) (Figure S6b). Moreover, patterns with a line width of 40 μm showed a very low hysteresis of 0.01% from start to end of the measurement. This shows that narrower lines exhibit greater sensitivity to applied strain, while broader lines have greater robustness and cycle stability.

While the extant literature on MXene-based strain sensors consists of planar films and hydrogels rather than micropatterns, the bubble printed Ti_3_C_2_T_X_ MXene patterns reported herein compare favorably in terms of their response to tensile strain. Notably, gauge factors (GF) between 5.15 and 0.08 for bending under tensile strain and 0.07 under compressive strain (calculated from Figure S7b) were obtained herein, with a high durability of 1000 cycles at a bending angle of 60.3° in both directions. In comparison, Ti_3_C_2_T_X_ MXene embedded in a polymer hydrogel gave a GF of 5.16 and could sustain 400 cycles at 50% strain with a response of 10%, around 2 times lower than the response measured herein.^21^ Comparing the response of bubble printed MXene micropatterns to compressive strain (<3%) with freestanding three-dimensional graphene/MnO_2_ composite networks (<1%), the bubble printed MXene patterns showed nearly 3 times higher sensitivity.^48^

Although the strain sensors reported herein are based on MXenes deposited onto PET films, their printing onto viscoelastic polymers may further enhance their sensitivity. Sensors based on graphene-carbon paste sandwiched between Ecoflex layers demonstrated high gauge factors up to 1.8 million across a broad strain range of 0%–625%, albeit with a durability of only 60 cycles.^49^ On the other hand, Ti_3_C_2_T_X_ MXene and carbon nanotubes deposited in a polydimethylsiloxane sponge exhibited a change in resistance around 50–70% for 60° bending, with a GF of 1939, and durability over 10000 cycles at 20% strain.^50^ A composite of Ti_3_C_2_T_X_ MXene/silver nanowires/thermoplastic polyurethane showed a GF of 33100 for stretching up to 120% strain over 1000 cycles, and exhibited a change of resistance of 11–14% for 60° bending under tensile strain.^22^ Ti_3_C_2_T_X_ MXene films deposited onto an elastomer showed a GF of 28629 at a strain of 0.6%, with high durability up to 5000 cycles.^51^

MXenes are prone to severe oxidative degradation, which can adversely affect their physical and electrical properties. This degradation is primarily due to oxidation of the surface when exposed to oxygen or water, converting metallic MXenes into their corresponding oxides. This poses a significant challenge to the long-term stability and performance of MXene-based devices.^52−54^ To investigate the extent of this effect, we conducted additional experiments focusing on the stability of MXene patterns printed with a line width of 40 μm. After a period of 127 days in ambient air, we observed that the samples exhibited an increase in resistance by a factor of 1.6 to 4.8 (Figure S8a), while retaining linear *IV* characteristics (Figure S8b). On the other hand, immersion of the substrates in water for 7 days led to significantly degradation, with the loss of linear *IV* characteristics observed for one sample, while two other samples increased in resistance by 2.1 and 2.9 fold (Figure S8c). While these results show that these sensors retain their function under prolonged air exposure, applications involving wet or moist environments should incorporate strategies to enhance their durability against oxidative degradation.^55−57^

In summary, the bubble printing of MXene-based patterns has been successfully demonstrated on a flexible substrate for the first time, allowing direct optical micropatterning of conductive MXene patterns on PET substrates without prior functionalization or postprocessing steps. The resulting conductive MXene micropatterns demonstrated distinct electrical responses under compressive and tensile strains, where resistance decreased under compressive strain and increased under tensile strain, which is in agreement with standard responses of conductive films and bulk composites.^58^ The resistance change within a single bending cycle is crucial for understanding the future potential of such conductive patterns for applications, as large changes in resistivity are suitable for resistive strain sensing, whereas small changes are beneficial for applications in which electronic properties should be maintained despite bending, such as wearable electronics. Thus, the direction of bending, i.e. whether tensile or compressive strain is applied, has a large impact on the potential applications. The repeated bending of the patterns revealed that cycling stability under compressive strain was higher than under tensile strain, with an average change of 11.3% after 1000 cycles of 60° bending under compressive strain compared to 61.6% under tensile strain. In addition, narrower MXene lines provided greater response to applied strain, while broader lines exhibited improved mechanical robustness as shown by improved cycle stability.

The most common method for fabrication of devices based on MXenes is the deposition of MXene films.^59−62^ Nevertheless, MXene micropatterning has been achieved using various approaches. With microcontinuous liquid interface production (μCLIP) patterns of around 100–200 μm were achieved.^63^ Inkjet printing has garnered significant attention due to its simplicity and the capability to deposit materials with reasonable precision. However, its resolution is typically above 20 μm, and its necessity for low-viscosity inks and the challenges associated with issues such as nozzle clogging and the coffee ring effect pose challenges for producing homogeneous films without additives.^23,64^ The highest resolution for inkjet printing of MXene reported so far is 40 μm line width.^65^ Aerosol jet printing has a similar resolution as inkjet printing, but allows for slightly greater flexibility in ink viscosity ranging from 1 to 500 cps. Despite this, it still requires careful handling of ink rheology and atomization, which has the potential to complicate the printing process and increase system costs.^66,67^ Electrohydrodynamic printing, on the other hand, offers significantly higher resolution, capable of reaching sub-100 nm levels (although printing of nanoparticles has only been achieved down to 10 μm). However, it faces practical challenges such as nozzle clogging, the need for conductive substrates, and potential damage to the substrate due to variable electric fields, while the fragility of the print tips can impede consistent production.^68,69^

There are a variety of emerging optical techniques which show promise for the printing of MXenes, such as opto-thermophoretic printing, however these are in a nascent stage of development and necessitate specific colloidal conditions, making their generalizability across various substrates and particles a substantial challenge.^70−72^ Bubble printing eliminates many of the common drawbacks associated with inkjet and aerosol jet techniques, such as rheology dependence, nozzle clogging, and the need for specific substrates. Bubble printing offers several advantages which make it a promising technique for micropatterning of MXenes, such as speed, simplicity, no need for pre/postprocessing steps, resolution down to 1 μm, and the capability for free-form patterning. Its straightforward setup makes it a compelling alternative for rapid, large-area patterning tasks where traditional methods may encounter limitations.^39^ Although the pattern size herein was limited by the size of the imaging spacers used, there is no real limitation, and printing of patterns in parallel can easily be achieved through the use of beam splitters or digital mirror devices for improved throughput.

Overall, bubble printed MXene patterns largely demonstrate advantages in sensitivity and stability for MXene-based strain sensors compared to previously reported sensor technologies, especially when considering the reduced dimensions of the micropatterns herein. This work not only serves as a proof of concept for printed microelectronics using a simple approach that eliminates the need for pre- or postprocessing, but also provides an understanding of how such patterns respond to tensile or compressive strain. The consistent electronic properties under compressive strain with bending angles up to 60° shows promise for applications in wearable and flexible electronics, whereas the high sensitivity to tensile strain in the range of 0 to 16° shows great promise for applications in resistive strain sensing including structural health monitoring and biomechanical sensing. Furthermore, the simplicity and versatility of this technique positions bubble printing of MXenes as a promising solution for advancements in structural health monitoring and wearable electronics.

## Supporting Information Available

Detailed methods, XRD spectrum of Ti_3_C_2_T_X_, additional SEM images of intact and damaged patterns and cross-sectional SEM images, *IV* characteristics for applied compressive strain, additional stress tests for tensile strain and response curves within a single cycle of bending for the first cycle and after 999 cycles, table of bending angles and their corresponding strain values, compressive strain tests for different line widths, and electronic properties and *IV* characteristics after exposure to air/water (PDF)
Video of bubble printing of Ti_3_C_2_T_X_ MXene on PET film (AVI)

## Supplementary Material

nl4c06355_si_001.pdf

nl4c06355_si_002.avi
