# Supplementary material for: Optically-Directed Bubble Printing of MXenes on Flexible Substrates toward MXene-Enabled Wearable Electronics and Strain Sensors
Source: Nano Lett. 2025 Apr 16;25(18):7258–65. doi: 10.1021/acs.nanolett.4c06355 (PMC12063168; doi:10.1021/acs.nanolett.4c06355)
Supplement: Supplementary file 1 — nl4c06355_si_001.pdf [file nl4c06355_si_001.pdf]

Supporting Information:

Optically-Directed Bubble Printing of MXenes on Flexible Substrates toward MXene-Enabled Wearable Electronics and Strain Sensors

Marcel Herber[†‡], Eric H. Hill[*†‡]
[†]Institute of Physical Chemistry, University of Hamburg, Grindelallee 117, 20146 Hamburg, Germany
[‡]The Hamburg Center for Ultrafast Imaging (CUI), Luruper Chaussee 149, 22761 Hamburg, Germany
*corresponding author: eric.hill@uni-hamburg.de

Video S1: Bubble printing of $Ti_3C_2T_X$ MXene on PET film.

Methods

Materials: Lithium fluoride (LiF, ≥ 99%) was obtained from Roth, titanium aluminum carbide 312 (Ti_3AlC_2, ≥ 90%, ≤ 40 µm) was obtained from Sigma-Aldrich, silver paste (Acheson 1415) was obtained from Plano GmbH, and hydrochloric acid (HCl, 37%) and isopropanol (IPA, ≥ 99.7%) were obtained from VWR Chemicals. Milli-Q water with a resistivity of 18.2 MΩ·cm was used for synthesis and cleaning of the product, as well as for the rinsing of the prepared samples.

$Ti_3C_2T_X$ MXene synthesis: The synthesis of $Ti_3C_2T_X$ nanosheets was carried out using the minimally intensive layer delamination method by Anasori et al.[1] To a PTFE bottle containing 80 mL of 9 M hydrochloric acid, 6.4 g of lithium fluoride was added under stirring (200 rpm) and was stirred for 5 minutes. Then, 4 g of Ti_3AlC_2 MAX phase powder was slowly added within 4 minutes. Afterwards, stirring was increased to 400 rpm. After 24 h the dark-black dispersion was washed with Milli-Q water by centrifugation (5 minutes per cycle, 3240 rcf) until the dispersion reached a neutral pH. The swelled MXene clay was redispersed in Milli-Q water and vortexed for 30 minutes, followed by sonication in an ice bath for 15 min under N_2 flow, and then centrifuged for 30 minutes at 2380 rcf. The collected supernatant had a dark-green color and a MXene concentration of 33.9 mg/mL. This showed a plasmon band at 741 nm (**Figure 1a**), while X-ray diffraction (XRD) showed the typical (00*l*) planes (**Figure S1**).

Bubble printing: First, a MXene dispersion containing 50% isopropanol, was prepared by mixing 5 µL of aqueous MXene dispersion (33.9 mg/mL), 35 µL Milli-Q water and 40 µL isopropanol, with a final MXene concentration of 2.12 mg/mL. The mixture was sonicated and vortexed for 5 seconds. A 0.12 mm imaging spacer (SecureSeal, Grace Bio-Labs, USA) was securely attached to a glass cover slip (Thickness 1). Following this, 15 µL of the as prepared MXene dispersion was pipetted into the well, which was then covered with a 50 µm thick polyethylene terephthalate (PET) film (CMC Klebetechnik GmbH, Germany) and covered with another glass cover slip. The substrate was then flipped and placed on the stage of an inverted microscope. A 532 nm continuous wave laser (Torus 532, Laser Quantum, UK) was then directed through a 5× beam expander (GBE05-A, Thorlabs, USA) prior to entering the microscope (Eclipse Ti2-A, Nikon, Japan). The laser beam was reflected by a 532 nm dichroic beam splitter and focused onto the interface between substrate and dispersion (spot size ~0.7 µm) with a 60× air objective (numerical aperture (NA) = 0.85, CFI Plan Fluor, Nikon). Under transmitted light, the sample was imaged with a CCD camera through a 533 nm notch filter (NF533-17, Thorlabs, USA) that prevents the laser light from passing through, as well as a 435 - 500 nm bandpass filter (FGB7, Thorlabs, USA) that blocks the fluorescence of the PET film from reaching the camera. A motorized microscope stage (H117P1NN, Prior Scientific Instruments, UK) was used for the movement of the laser beam. The samples were printed using an optical chopper system (MC2000B-EC, Thorlabs, USA) with a chopper blade (MC1F10HP, Thorlabs, USA) at 5000 Hz and a laser power of 27.7 to 49.5 mW (typically 49.5 mW) was used. After the printing process, the top cover slip was taken off, and the substrate was rinsed with Milli-Q water and briefly dried under compressed air. A graphic of the optical setup is shown in **Figure 1b**.

Resistance measurements: Bubble printed MXene lines were conducted with silver paste to connect the samples to a source measure unit (2601B-PULSE, Keithley Instruments, USA). Current-voltage characteristics of the samples were measured in a range of −5 to 5 V, and resistance measurements were performed with an applied bias of 5 V and a current limit of 10 mA.

Bending setup: Bending experiments were performed on a custom-built bending setup (**Figure 3a**). The bending axis with a bending radius of 2 mm is connected to a stepper motor (TS3079N535, Tamagawa Seiki Co., Japan), which allows rotation of the bending platform with a resolution of 0.9°. To control the stepper motor a commercial driver board (SBC-Motodriver3, SIMAC Electronics GmbH, Germany) was connected to a micro controller (Arduino Mega 2560, Arduino, Italy). A light sensor was used to properly return the bending platform to the initial position before every measurement. Custom Python scripts were used for data acquisition and to control the movement of the setup. The strain on the bent PET films was calculated by taking photographs at various bending positions. Circles were then fitted around the curvature of the films to determine the radius of curvature (r). Subsequently, the length between two fixed points on the films was measured to obtain any change in length due to bending. The surface strain (ε_S) was subsequently calculated using the following formula: $\varepsilon_S = h/(2r)$, where h is the thickness of the PET film.[2] This formula provides the strain at the film surface. The applied strain was determined by calculating the ratio of the change in length (ΔL) to the original length (L) of the film, denoted as $\Delta L/L$.[2] The values for strain obtained by image analysis were smoothed with the Savitzky-Golay method.

Characterization: UV-Vis spectroscopy was performed on a Cary 60 (Agilent Technologies, USA). X-ray diffraction (XRD) was measured with Cu-K_α radiation (1.54 Å) on a PANalytical X'Pert MPD diffractometer (Philips, Netherlands). Transmission electron microscopy (TEM) images were taken on a JEOL JEM 1011 (JEOL, Japan) with an acceleration voltage of 100 kV, and scanning electron microscopy (SEM) images of the bubble printed lines were taken on a Zeiss LEO Gemini 1550 with an acceleration voltage of 2 kV (Carl Zeiss Microscopy Deutschland GmbH, Germany).

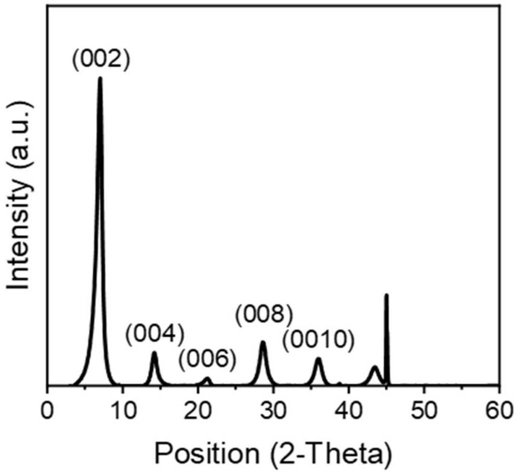

Figure S1. X-ray diffraction (XRD) spectrum of $Ti_3C_2T_X$ MXene.

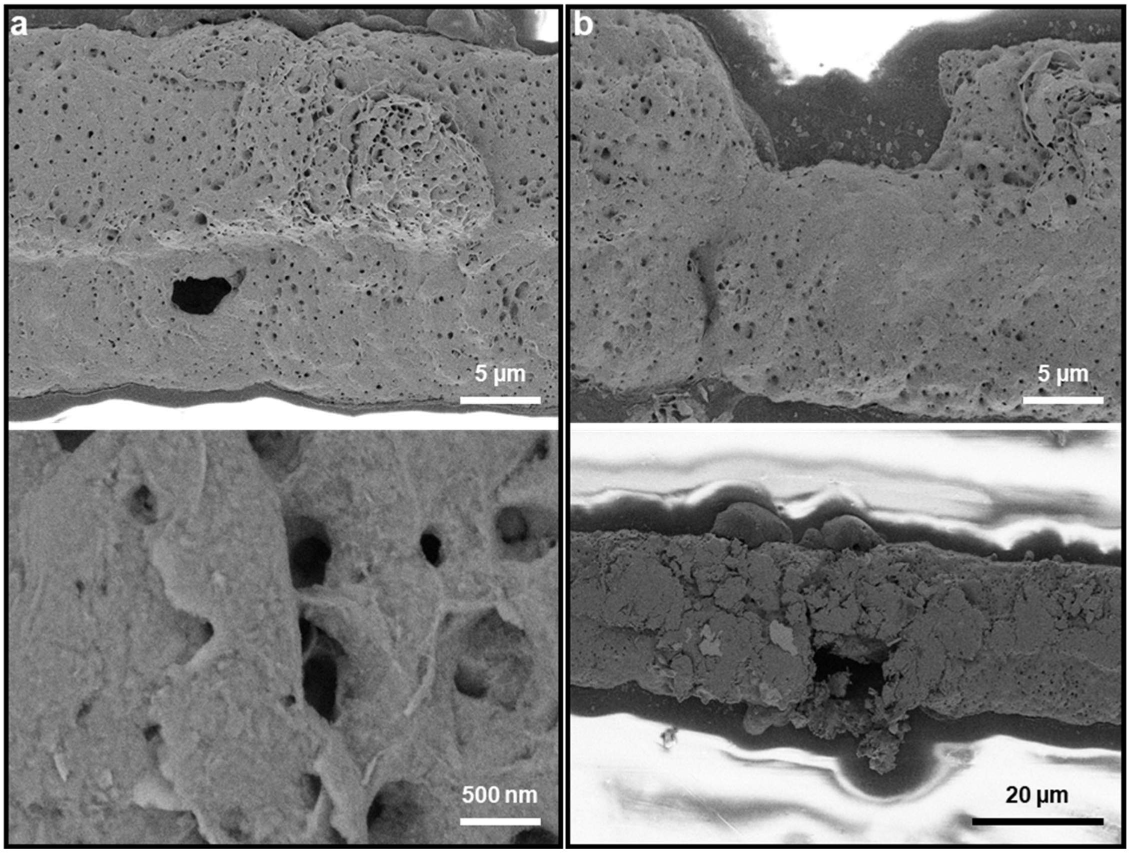

Figure S2. Scanning electron microscopy (SEM) images of bubble printed MXene patterns (a) without applied strain and (b) after 1000 applied to cycles of bending to 60.3º under tensile strain. The lack of conductivity of the PET films leads to the significant charging observed away from the MXene line.

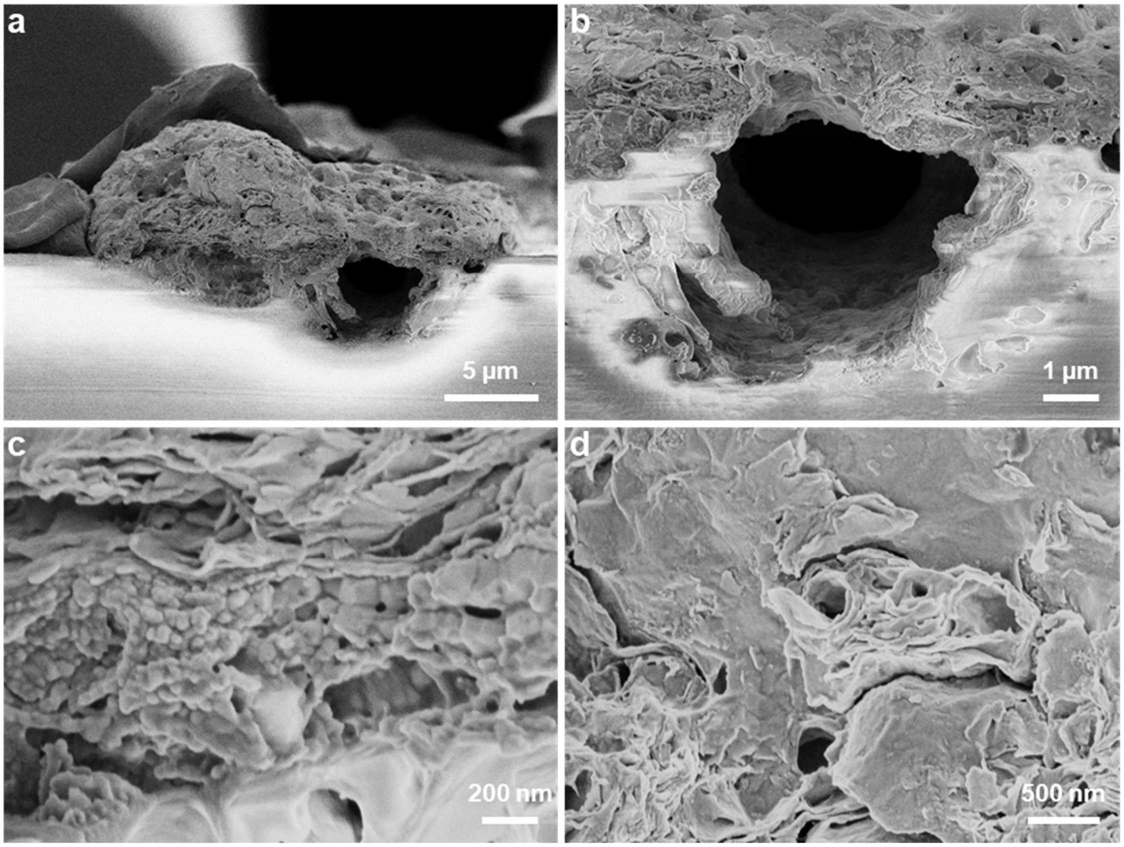

Figure S3. Cross-sectional scanning electron microscopy (SEM) images of bubble printed MXene patterns.

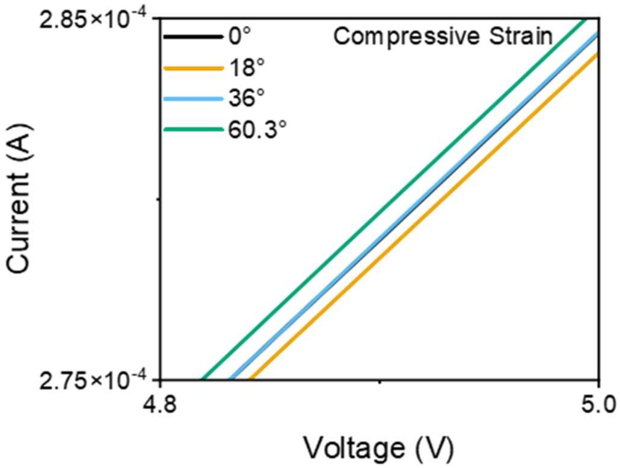

Figure S4. Zoom of *IV* curves of MXene patterns at different bending angles for applied compressive from **Figure 2c**.

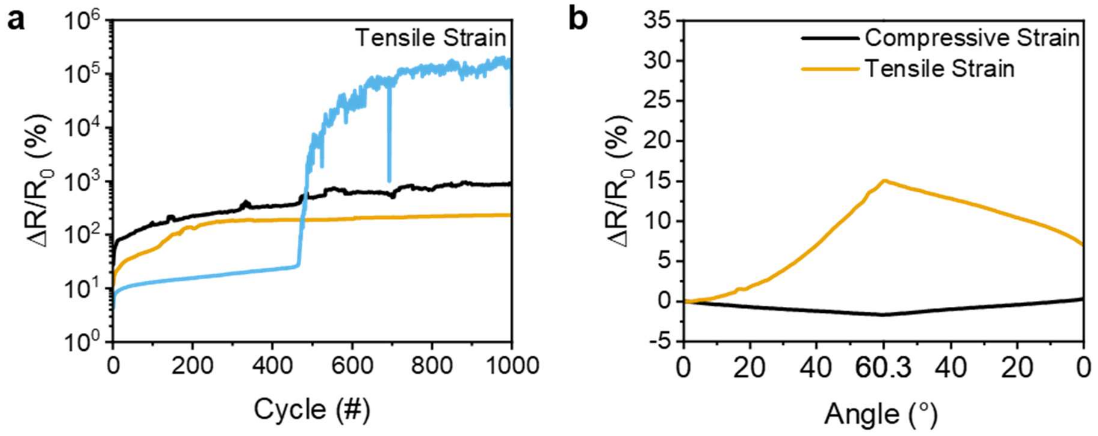

Figure S5. (a) MXene patterns bent to 60.3º under tensile strain which show evidence of partial failure (b) Sensitivity/response tests within a single bending cycle for the first bending from **Figure 4**.

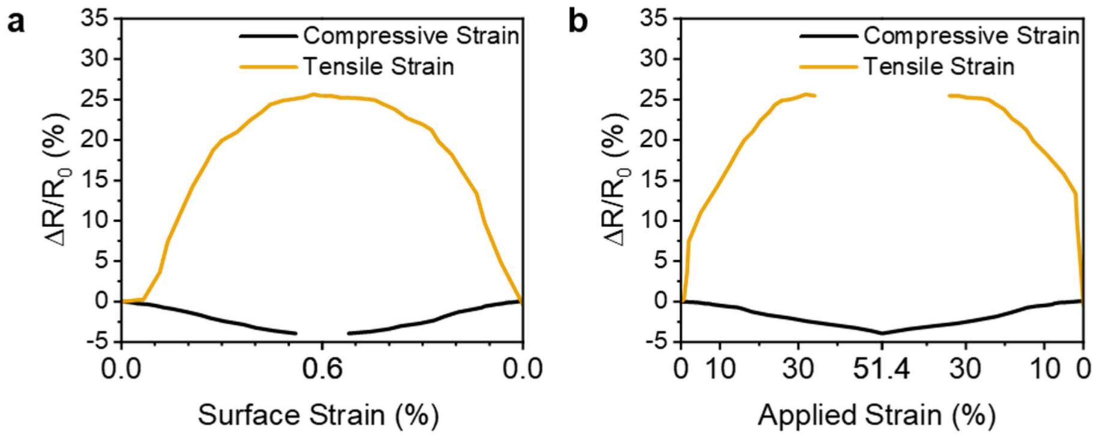

Figure S6. Average change in resistance as a function of (a) surface strain and (b) applied strain within a single cycle of bending to 60.3° after 999 cycles.

Table S1. Bending angles and their corresponding strains.

Tensile Strain			Compressive Strain		
**Bending [°]**	**Surface Strain [%]**	**Applied Strain [%]**	**Bending [°]**	**Surface Strain [%]**	**Applied Strain [%]**
0.0	0.000	0.000	0.0	0.000	0.000
2.7	0.065	0.730	2.7	0.018	2.590
5.4	0.113	1.615	5.4	0.033	4.675
8.1	0.136	1.964	8.1	0.047	6.110
10.8	0.174	5.054	10.8	0.059	7.488
13.5	0.209	9.260	13.5	0.079	7.948
16.2	0.248	12.664	16.2	0.095	9.702
18.9	0.269	14.616	18.9	0.112	12.104
21.6	0.296	16.141	21.6	0.122	14.426
24.3	0.340	18.292	24.3	0.149	15.843
28.8	0.377	20.350	28.8	0.191	18.161
33.3	0.413	22.605	33.3	0.232	22.331
37.8	0.436	24.034	37.8	0.261	27.676
42.3	0.475	25.774	42.3	0.303	32.292
46.8	0.500	27.989	46.8	0.359	38.324
51.3	0.537	29.759	51.3	0.395	44.495
55.8	0.565	31.891	55.8	0.449	48.628
60.3	0.587	34.186	60.3	0.512	51.365

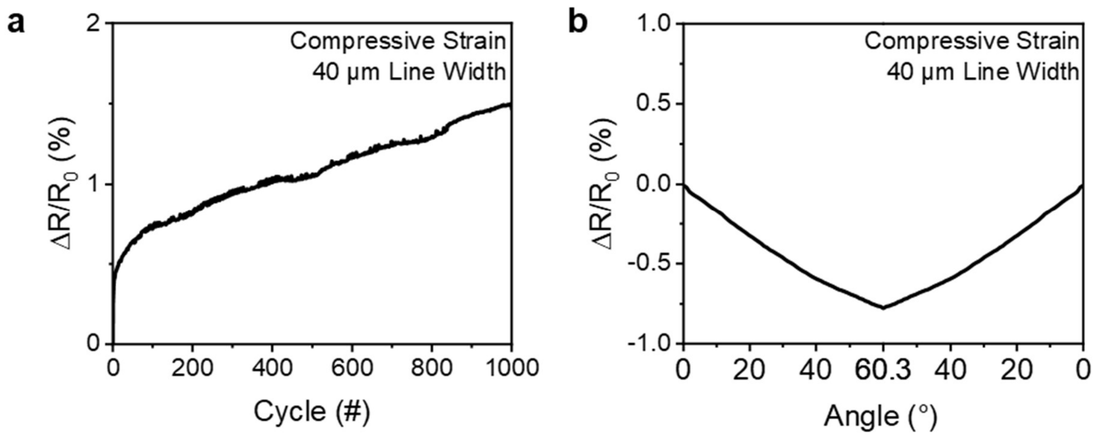

Figure S7. (a) Cycling stability curve and (b) sensitivity/response curve of a single bending cycle of averaged MXene patterns with a line width of 40 μm bent to 60.3º under compressive strain.

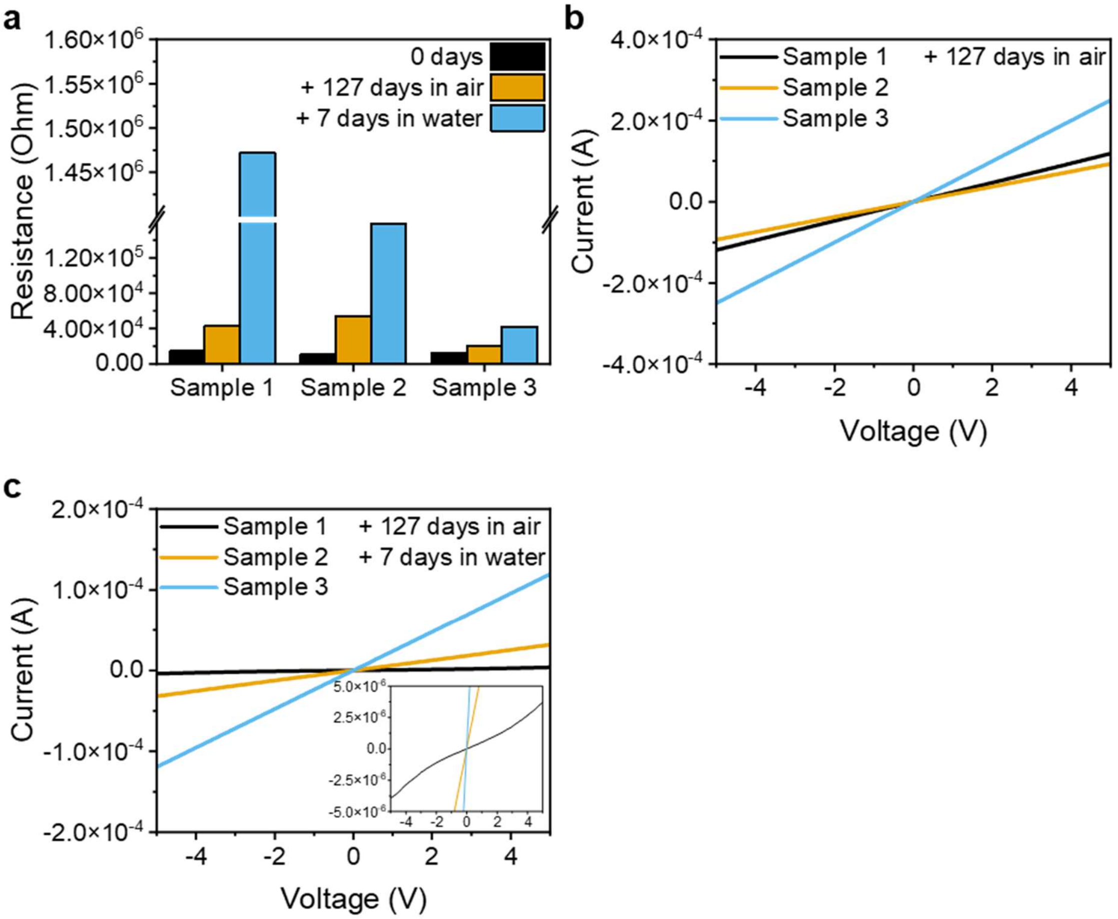

Figure S8. Stability of samples with a line width of 40 µm upon exposure to air and water. (a) Comparison of sample resistance after 127 days in ambient air followed by an additional 7 days submerged in water. Corresponding *IV* curves are depicted for (b) samples after 127 days of air exposure, and (c) samples after 127 days of air exposure followed by an additional 7 days of water immersion.

References

(1) Thakur, A.; Chandran B.S., N.; Davidson, K.; Bedford, A.; Fang, H.; Im, Y.; Kanduri, V.; Wyatt, B. C.; Nemani, S. K.; Poliukhova, V.; Kumar, R.; Fakhraai, Z.; Anasori, B. Step-by-Step Guide for Synthesis and Delamination of Ti3C2Tx MXene. *Small Methods* **2023**, *7* (8), 2300030. https://doi.org/10.1002/smtd.202300030.

(2) Kuwahara, K.; Taguchi, R.; Kishino, M.; Akamatsu, N.; Tokumitsu, K.; Shishido, A. Experimental and Theoretical Analyses of Curvature and Surface Strain in Bent Polymer Films. *Appl. Phys. Express* **2020**, *13* (5). https://doi.org/10.35848/1882-0786/ab8346.
